# Suitability of GWAS as a Tool to Discover SNPs Associated with Tick Resistance in Cattle: A Review

**DOI:** 10.3390/pathogens10121604

**Published:** 2021-12-09

**Authors:** Nelisiwe Mkize, Azwihangwisi Maiwashe, Kennedy Dzama, Bekezela Dube, Ntanganedzeni Mapholi

**Affiliations:** 1 Agricultural Research Council-Animal Production Campus, Private Bag X2, Irene 0062, South Africa; norman@arc.agric.za (A.M.); dubeb@arc.agric.za (B.D.); 2 Department of Animal Sciences, University of Stellenbosch, Private Bag X1, Matieland, Stellenbosch 7602, South Africa; kdzama@sun.ac.za; 3 Department of Agriculture and Animal Health, University of South Africa, Private Bag X6, Florida 1710, South Africa; maphon@unisa.ac.za

**Keywords:** tick control, genotyping technology, quality control, association test

## Abstract

Understanding the biological mechanisms underlying tick resistance in cattle holds the potential to facilitate genetic improvement through selective breeding. Genome wide association studies (GWAS) are popular in research on unraveling genetic determinants underlying complex traits such as tick resistance. To date, various studies have been published on single nucleotide polymorphisms (SNPs) associated with tick resistance in cattle. The discovery of SNPs related to tick resistance has led to the mapping of associated candidate genes. Despite the success of these studies, information on genetic determinants associated with tick resistance in cattle is still limited. This warrants the need for more studies to be conducted. In Africa, the cost of genotyping is still relatively expensive; thus, conducting GWAS is a challenge, as the minimum number of animals recommended cannot be genotyped. These population size and genotype cost challenges may be overcome through the establishment of collaborations. Thus, the current review discusses GWAS as a tool to uncover SNPs associated with tick resistance, by focusing on the study design, association analysis, factors influencing the success of GWAS, and the progress on cattle tick resistance studies.

## 1. Introduction

Traditionally, animal improvement programs were limited to phenotypic information only, which may be ineffective for traits that are costly to measure, such as tick resistance. In cattle production, the presence of bovine ticks is considered as one of the main sources of diseases, which are detrimental to animal health [1]. Ticks and tick-borne diseases (TTBDs) have substantial effects on animal health and welfare wellbeing, as well as a serious economic impact in both developed and developing countries [2]. To mitigate the bovine tick burden, a wide range of tick control strategies have been adopted; however, they are ineffective in completely eradicating ticks. Currently, farmers use acaricides, which were efficient when they were introduced; however, they later developed limitations which are detrimental to the animal production economy and to consumers. Various researchers have reported that the prolonged utilization of acaricides on food-producing animals creates the development of acaricide resistance by ticks, subsequently reducing the efficacy of chemicals [3,4]. Additionally, acaricide residues have been traced in milk and meat products [5]. The presence of these residues in food products and in the environment poses a health threat to human beings. This underlines the need for alternative tick control measures, which are chemically-free and environmentally-friendly [6]. A potential alternative approach to control ticks would be the use of genomic information, which entails the exploitation of genetic variation in host resistance to tick infestation. The success of this approach depends on the discovery of genetic determinants associated with low tick load in cattle. 

The development of high throughput genotyping technologies has provided an opportunity to identify novel genetic variants, such as single nucleotide polymorphisms (SNPs), associated with economic traits in cattle. SNPs are genetic markers of choice because they are heritable and abundantly distributed across the genome. Genome-wide association studies (GWAS) are increasingly becoming the common experimental approach to investigate SNP markers associated with various economic traits in animal production. This approach operates by associating the phenotype with the genotype data to investigate the causal genetic variants for traits of interest using statistical models. The use of SNP markers in breeding for tick resistance (low tick load in cattle) has the potential to assist breeders in making informed decisions to improve host resistance to ticks in cattle [7,8,9]. Through the use of GWAS, a number of studies have been conducted to investigate genetic variants for tick resistance in cattle, in different breeds and regions [10,11,12,13,14]. 

Studies that have been conducted to date have presented evidence of the association of various genomic regions with low tick load in cattle and recommended the validation of the discovered regions. Some of the challenges associated with GWAS include different phenotyping methods and genotyping strategies. These challenges could be overcome by the standardization of phenotyping procedures for tick count [15]. Genotype imputation has been identified and recommended as a cost-effective approach to account for the missingness of genotyped data and facilitate the improvement of GWAS power. Additionally, the establishment of collaborations holds the potential to solve issues associated with small discovery populations and running costs for GWAS for tick resistance. 

Despite the number of tick resistance GWAS studies that have been conducted to date, the availability of data is still a challenge, which is a global challenge hindering the success of improving tick resistance traits through genomic selection. Several researchers noted that in depth knowledge of genome variation for tick resistance in cattle is required [7,16]. The generation of genomic information for host resistance to ticks is currently gaining more attention because there is a need for the knowledge on genetic determinants influencing this trait. This paper reviews GWAS as a genetic tool to identify genetic variants associated with resistance to ticks in cattle.

## 2. GWAS Overview

The advent of high density SNP genotyping platforms has provided opportunities to detect quantitative trait loci (QTLs) and uncover the genetic architecture of quantitative traits. This development has stimulated interest among researchers to explore genetic variabilities associated with various diseases affecting animals using GWAS. Genome-wide association analysis relies on recombination to rearrange the genome. Its underlying principle is to seek correlation between phenotype and genotype based on a non-random association of alleles at two or more loci [17,18]. 

This GWAS approach has successfully uncovered genetic determinants associated with disease susceptibility and resistance in humans, animals, and plants [19,20,21] However, using this approach to uncover genetic determinants associated with traits which follow polygenic patterns of inheritance, such as tick resistance, is not straight forward, since such traits are controlled by multiple genes. Despite this challenge, the approach is used to search for marker variants indirectly associated with certain diseases or traits of interest by assuming that a marker is in linkage disequilibrium (LD) with the underlying causal variant [22]. Linkage disequilibrium refers to the non-random association of alleles at different loci in a given population [23]. Currently, the GWAS approach is gaining popularity in mapping QTLs associated with traits of economic importance or complex traits. This is because GWAS is able to detect variants that can be in LD with the causal variant, and this information could be used to narrow genomic regions that harbour causal variants [24,25], providing genetic determinant information that could be useful for the genetic selection of economic traits, such as tick resistance in cattle. The continued success of GWAS depends on careful population selection and collaborative analytical approaches. Work by [26] reviewed the guidelines for successful GWAS analysis intensively and presented a useful workflow, which might be of value when conducting GWAS. 

Bovine GWAS have successfully discovered genetic determinants associated with distinctive disease resistance or susceptibility, such as tuberculosis [27], resistance to ticks [8,12], mastitis [28]; and foot and mouth disease [29]. Moreover, this approach has been used in the successful mapping of genetic variants associated with meat quality [30,31] and milk production [32,33]. Such studies assist in providing information on the genetic architecture of QTL, generate biological knowledge about the expression of economic traits and facilitate the improvement of genomic selection.

## 3. Computer Software for GWAS and Genomic Public Databases

The most commonly used computer programs for GWAS are presented in Table 1. They perform the same activities and their access is generally free. The effective use of these programs requires the user to have operating skills.

**Figure 1:**
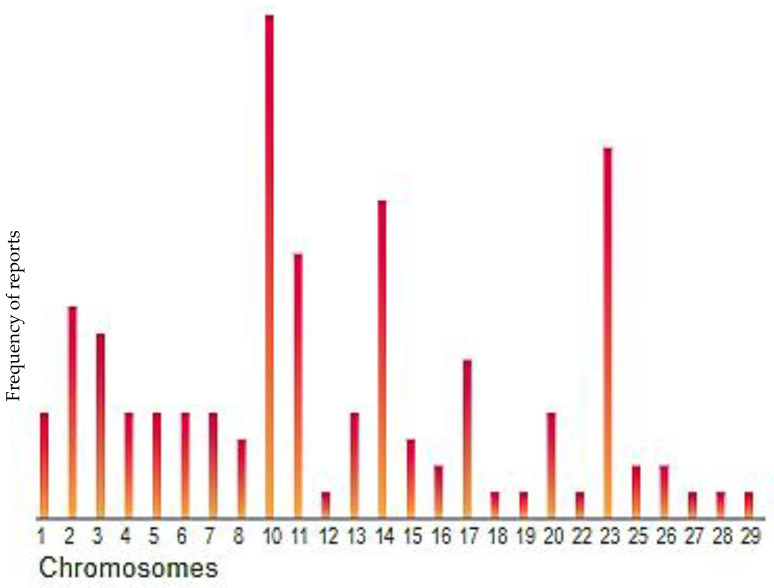
Distribution of tick resistance related QTL/associations in bovine, based on count of report data (sourced from: https://www.animalgenome.org/cgi-bin/QTLdb/BT/index (accessed on 4 September 2020).

The information generated from genomic studies is housed on different web databases for public access. Such databases include NCBI, EMBL-EBI, Ensembl, Animal QTLdb and NAGRP. Some of these databases are not specific to any organism, while some are specific to livestock genomics (Table 2). To date, a number of QTLs and associations related to tick resistance have been identified in different cattle breeds using different research approaches. The information of the QTLs and associations is recorded in a database known as Animal QTLdb, which is currently updated whenever there is an availability of new information [40]. Based on the available information, it is noted that most of the QTLs and associations have been uncovered mainly on chromosome 10, followed by BTA23, BTA14, BTA11, BTA2 and others. This is depicted in Figure 1, sourced from the Animals QTLdb database. Information on African indigenous breeds is lacking on these databases. The limited information contained on these databases concerning tick resistance in cattle breeds shows that more research still needs to be conducted, especially in African cattle breeds.

## 4. Available Genotyping Platforms and Coverage

The abundance of SNPs in the genome and its ability to be amenable to high throughput automated analysis make SNP genotyping the most preferred approach to studying genetic variation in animals, humans, and plants [41,42,43]. Moreover, SNPs are heritable and allow single base resolution, making the identification of causal markers easy [44]. Initially, SNP genotyping arrays were developed for human studies [45], and then, the technology was adopted in animal and plant research. In cattle research, three well known commercial companies that produce commercial Bovine SNP arrays are Illumina, Neo-Geneseek^®^ and Affymetrix. These companies have developed SNP genotyping platforms with different densities, which are used for GWAS, identification of selection sweeps, and investigating genome-wide genetic diversity and relationships in cattle. The summary of SNP genotyping platforms available for Bovine is provided by [46]. The SNP genotyping platforms include different densities of SNPs, ranging from as low as Golden Gate Bovine 3K (2900 SNPs) to Bovine HD (777,962 SNPs) [47]. The density of an array plays an important role in the success of a GWAS study. It was recommended by [48] that denser SNP arrays should be used for crossbreed GWAS studies, due to its large hypothetical effective population size. GWAS experiments performed using data from denser SNP arrays are provided with enough marker density to dissect the genetic architecture of the trait of interest. In instances where low density SNP arrays have been used, genotype imputation is advisable. This phenomenon will be briefly discussed later in this article. 

The development of SNP arrays is advancing rapidly, making genomic selection feasible [47]. They noted that the development of genotyping platforms has not been regulated under a standardized system. This results in difficulties in making comparisons and merging data genotyped by different commercial companies. Therefore, there is need for standardization during SNP array development to minimize downstream research challenges [47]. Despite this success, studies on local African breeds are still facing the challenge of the SNP arrays being originally created using exotic breeds. This underscores the need for the development of SNP arrays that will incorporate ancestry data from African breeds. The development of such SNP arrays could potentially extend insights on the architecture of traits being investigated using African cattle populations. Although the decreasing cost of genotyping potentially makes GWAS possible and affordable, in Africa the cost of genotyping is still relatively expensive. This is a challenge for GWAS because it is difficult to genotype the minimum number of animals recommended [48]. The minimum number of animals required is determined by conducting a statistical power test, to ensure the low rate of discovering false positive results in GWAS [49]. The issue of attaining a better number of samples for GWASs can be solved through the establishment of collaborations. 

## 5. Testing for an Association

The main goal for GWAS is to test a null hypothesis, stating that there is no association between the genotype and the expression of the trait of interest. This is facilitated by choosing the appropriate association test approach, generally influenced by covariates, population structure, study population and pedigree structure [50].A single locus statistical test and multiple locus tests are the two approaches that are currently being used, depending on the focus of the study. A single locus statistical test compares the genotype and the phenotype by focusing on one SNP at a time [51]. This test uses a regression model, assuming that the trait being studied is normally distributed and the variance is the same within a population. Testing one SNP at a time results in multiple tests, which may produce false positive and false negative results [48,52,53]. Therefore, it is necessary to correct for multiple testing to prevent spurious associations, making it impossible to conduct follow up studies. This can be performed using the false discovery rate (FDR) and Bonferroni correction. The adjustment through the FDR approach corrects for the expected rate of false discoveries, and also gives the investigator an insight into the proportion of true associations in the study [54,55] However, this adjustment is considered less stringent compared to the Bonferroni correction [55,56].

After the adjustment, a SNP is considered statistically significant if its p value is less than or equals the adjusted genome-wide cut off [55]. A single SNP association approach worsens the missing heritability problem, which is the gap between the heritability measured using pedigree information and that measured through GWAS [57,58]. Biological mechanisms, such as epistasis, epigenetics and others, are attributors of missing heritability. However, in a human-based study, none of these mechanisms accounted for the missing heritability [59] The study included human microbiome information to understand the heritability of a given trait in humans. They reported that microbiome is associated with many important traits and encodes for extra genes which interact with human genes. The interaction can be a source of variation and phenotypic plasticity [59]. Thus, the inclusion of microbiome in GWAS for cattle can be used to solve the issue of missing heritability. Alternatively, the multilocus association approach can be adopted, because it examines nonlinear relationships genome-wide. Multilocus models do not require Bonferroni correction; this is beneficial because it reduces the high chances of losing many loci associated with the targeted trait through failing to meet the stringent requirement for the significant test, as it happens in single locus models [60]. Multilocus models are more advantageous compared to single locus models, because of their ability to allow the estimation of three variance components, a high power of QTL detection, and the SNP effect is random [61]. Table 3 shows some examples of single and multilocus models that are currently being used in GWAS across different species. 

**Figure 2:**
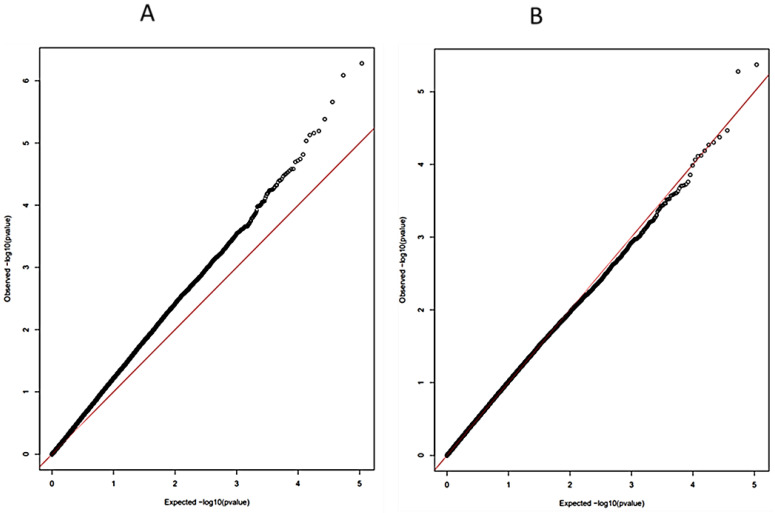
(**A**) Quartile–quantile plots before correction. (**B**) Quartile–quantile plots after correction.

During the association analysis, population stratification is regarded as one of the confounding effects that can inflate the variance of the usual statistics [65]. The inflation of test statistics may potentially attribute high false positive discoveries; hence, it should be accounted for. There are two ways to account for population stratification in GWAS: the variance is adjusted using genetic control or principal component analysis. It was pointed out by [65] that genomic control adjusts the variance by calculating the statistics on data from null loci. Figure 2 shows the quartile–quantile plots before correction (A) and after (B). Figure 2A shows a departure from the diagonal observed, which indicates a high inflation rate. Figure 2B shows the improvement after correction using genomic control, where the inflation rate has decreased.

At the end of the association analysis, an independent association test replication is recommended on the SNPs to validate the findings [66]. This is, however, still not applicable for most studies, because of the associated cost implications, time, and other factors associated with the study design. When a replication study is conducted, GWAS is performed on statistically significant SNPs. This process makes use of a small sample size, attributing to a low GWAS power and making it difficult to confirm the initial findings [68]. Lack of replications does not necessarily mean findings are not valid, but the study should be properly designed, and all confounding factors accounted for, to ensure the validity of the findings.

**Figure 3:**
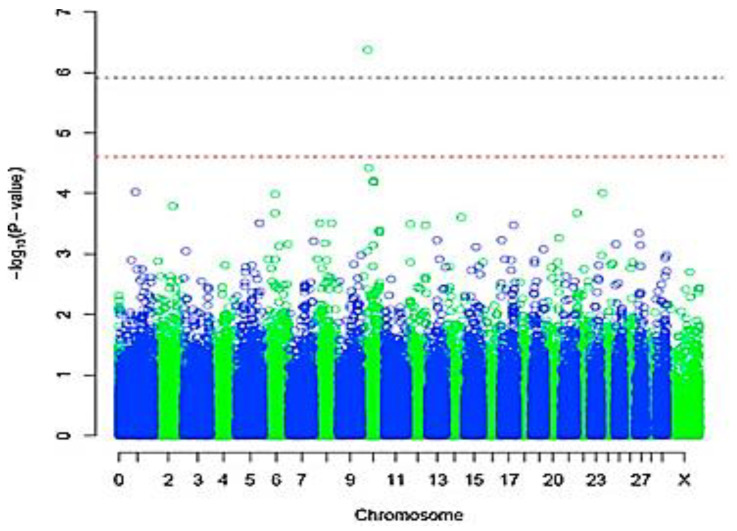
Manhattan plot showing findings for a single marker GWAS where the association of low tick load (total *A. hebraeum* ticks) and genotype was assessed in Nguni breed, using a genome-wide *p* value < 0.05 as a cut-off. The redline indicates suggestive threshold and the grey indicates the genome-wide cut off (taken from [8]).

**Figure 4:**
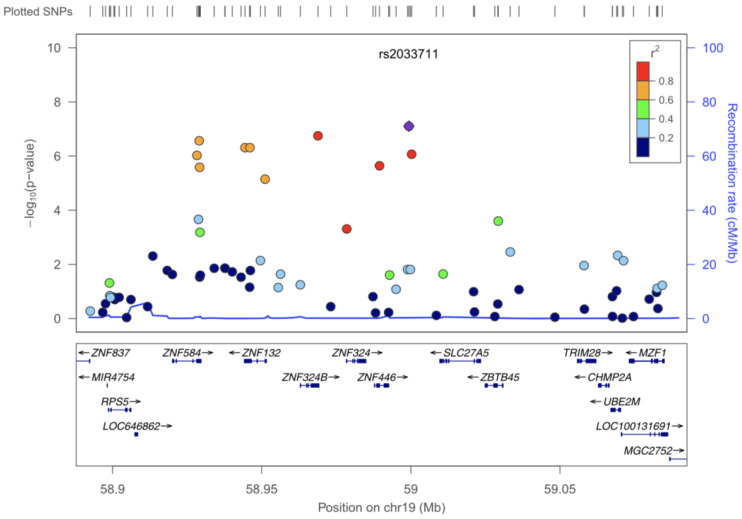
LocusZoom plot showing in depth findings for most significant SNPs in Chromosome 19, from a human GWAS that was focused to study genetic variation underlying renal uric acid excretion in Hispanic Children [75].

The preferable mode to summarize and present GWAS findings visually is the use of a Manhattan plot (Figure 3). Figure 3 presents y-axis −log_10_ (*p*-value) versus x-axis (the chromosome position for each SNP tested), where each circle signifies a SNP. Generally, the SNPs are stacked together to form a signal that is influenced by the level of LD amongst the SNPs with relation to the causal marker. Several studies have used Manhattan plots to present their GWAS findings in cattle [8,14]. In South Africa, [8] conducted a GWAS study on the indigenous Nguni and produced the plots presented in Figure 3. The two dotted horizontal lines depict the suggestive (red) and the actual genome-wide cut off line [grey], which represents the level of significance. If a SNP passes the grey line, it is considered significant. On this study, a significant SNP associated with tick resistance in Nguni cattle was observed in BTA 10. There are many free tools that can be used to graphically plot a GWAS Manhattan diagram; examples include SNPEVG [69], R package (qqman) [70], Stata [71], and Manhattan ++ [72]. When a GWAS association has been detected, tools such as Manhattan harvester [73] and Locus Zoom [74] provide opportunities to study the detected region in depth. Both tools focus on the physical position of the chromosome of interest; however, LocusZoom is more informative because it allows the visualization of LD levels, recombination rates and genes [74]. The LocusZoom tool has mostly been used in human research. Figure 4 depicts an example of a LocusZoom plot, sourced from a human GWAS study [75], showing the architecture of a region of interest in chromosome 19 (significant SNPs, associated recombination rate, and genes). The use of tools such as LocusZoom in bovine research will provide an in-depth insight into the landscape of genetic contribution associated with the expression of economic traits, such as tick resistance, growth traits, milk production and others.

## 6. Post GWAS Analysis

The discovered GWAS hits are used to physically map candidate genes underlying the trait being studied. The National Centre of Biotechnology Information (https://www.ncbi.nlm.nih.gov/ accessed on 6 December 2019) and ENSEMBL(http://www.ensembl.org/index.html accessed on 6 December 2019) databases are generally used for gene annotation to identify candidate genes associated with identified SNP markers [76]. When the genes have been identified, their biological functionality or relevance can be verified through the use of functional annotation databases such as DAVID and KEGG [58,77,78]. Furthermore, it is also possible to create gene networks using open source software such as Cytoscape [79]. The gene network provides a better biological understanding of the interaction of genes underlying the trait of interest. A recent GWAS by [14] identified SNPs and candidate genes (*TREM1, TREM2, CD83, MYO5A, TREML1, PRSS16*) associated with tick resistance, and then used the information to create a gene network. Tick resistance is a complex trait that is influenced by various determinants. Therefore, gene networks are very important to give insight on the interconnection of genes responsible for the expression of the tick resistance trait. Despite the success that has been made, the inferring of true causal genes and biological mechanisms from GWAS results in tick resistance studies is still a challenge. This is due to the difficulties associated with the interpretation of GWAS findings and the limitation of available data. 

## 7. Factors Influencing the Success of GWAS

### 7.1. GWAS Experimental Design

The success of GWAS requires proper study experimental design, in addition to factors such as population of interest, sample size and standardized data collection. In addition, concise pipelines to execute the actual analysis should be taken into consideration. Phenotyping data should be properly collected to reduce high rates of outliers, which may potentially create noisy data. The data is often tested for normality, to assess the distribution and the presence of outliers so that necessary steps can be taken to address the violation of normal distribution assumptions and the removal of outliers. Generally, the tick resistance trait is known to be not normally distributed and literature shows that some studies addressed this issue by transforming the data to confer normality. It has been noted that, although removal of outliers is important, it affects the size of the population tested. Selection of population for association analysis generates a structure that leads to specific genetic variation and an effect on the end use of association analysis [49]. In cattle, within family pure breeds and crossbreeds have been used for dairy and beef GWAS experiments to identify genomic regions associated with phenotypic variation in economically important traits. Crossbred lines, specifically the F2 design, have been identified as an appropriate design because they exhibit a high level of LD compared to pure lines. High LD potentially increases the power of GWAS [80]. Studies have been conducted to study the LD patterns within the crossbred F2 population [48,81,82]. 

In relation to cattle tick resistance studies, GWAS studies have been conducted using the crossbred F2 design in different regions [12,14]. However, it can be noted that some previous studies were conducted using data that was genotyped using microsatellite markers. The limitation of microsatellite markers involves the lack of an adequate number of informative markers, while on the other hand, SNPs are very abundant in the genome [83]. A study by [14] is currently the only known study that used the F2 crossbred design to discover tick resistance genetic determinants using SNP genotyped data. Although all these studies have been conducted, and information generated, the development of crossbred F2 populations remains a challenge. More time is required to build the required population, and this is associated with high costs of SNP genotyping a large sample size, especially in developing countries. However, there is need for this population design to be used for the genomic improvement of the tick resistance trait, regardless of the associated shortcomings. 

### 7.2. Phenotyping

Phenotyping for tick resistance in cattle is often assessed through natural or artificial infestation. The assessment of tick count under the approach of interest is one of the baseline indicators for tick resistance differences. Tick count is the commonly used mode of phenotyping in studies that assess tick resistance differences in a population. Counting ticks for GWAS phenotyping where a large population size is the requirement becomes a bottleneck. A study by [84] noted that counting ticks on every animal in a population is labor intensive, time consuming, and requires trained technicians and expensive infrastructure to constrain the animal. This stresses the animals through handling and the use of a trained technician does not rule out the possibilities of bias tendencies when counting. More challenges are faced under natural infestation because of multiple tick species and the need to categorize them accordingly when counting. All these shortcomings play a part in the reduced success rate of recording a tick count for big study populations. A study by [85] on Nguni cattle estimated the correlation between whole body tick count and tick count in different body parts. They also assessed correlations between tick species and different seasons, and observed that some tick species were prevalent in certain seasons, indicating that seasons are influential on tick distribution. The approach for this study is very informative because it gave insight into the distribution of ticks in different parts of the animals. The challenge is that the process of counting ticks is labor intensive, and this will be very difficult for studies with large population size. 

Artificial tick infestation has been used an alternative approach; however, this approach does not represent the true reflection of natural infestation in regions where various tick species inhabit [84]. To solve this problem, ref. [86] introduced a scoring method that overcame some disadvantages associated with tick count. However, ref. [87] reported that the scoring method provides a low heritability, as compared to tick count. Low heritability constitutes a reduced power to detect association, since heritability is a good indicator that explains the genetic variance contribution towards the expressed phenotype [49]. Therefore, since the scoring method is associated with low heritability estimates, tick count remains a better method of phenotyping for tick resistance GWASs. However, there is need for high throughput phenotyping methods that will eliminate the issues of biasness when counting ticks and reduce associated costs and stress imposed to animals.

### 7.3. Population Size

Population size is amongst the limiting factors affecting the statistical power of a GWAS. A small population size results in a reduced statistical power to detect a causal variant. This poses a major challenge in understanding the biological mechanism underlying economic traits such as tick resistance. Generally, a population comprising 1000 individuals is regarded as a better population size, constituting about 80% GWAS statistical power [88]. In certain research set ups, it is difficult to attain a minimum of 1000 samples to conduct a GWAS, because of various factors at play associated with infrastructure and costs. These factors are not limiting researchers’ drive to find information that could be used to improve animal welfare and facilitate sustainable food security in the current trying times. According to [89], a population of individuals ranging from 100 to 500 can be suitably used to perform GWAS. However, such a population needs to be carefully selected based on the status of the genetic variation of the trait being studied, and other factors influencing phenotype such as environment. Moreover, the selection criterion must take population stratification into consideration to avoid false positive results. The selected GWAS population should result in low stratification [90] and large genetic variation to potentially detect true variants that can be used in breeding schemes to improve the trait of interest. 

GWAS statistical power is defined as the probability of rejecting a null hypothesis under an assumed alternative hypothesis [88]. It depends, mainly, on a clear study design and the number of samples used for the analysis. Generally, one thousand samples, equating to 80% power, are considered the normal number in human and animal studies [88]. The same applies to livestock studies. However, the issue of sample numbers remains a problem in developing countries. Hence, many GWAS livestock studies are conducted on samples below one thousand. Factors playing a role in this regard include the lack of recorded phenotypes for certain traits and the availability of resources to cover genotyping costs. Although such costs are gradually decreasing, researchers in Africa are still battling to afford genotyping on cattle numbers near the normal one thousand mark. The situation is even more difficult when high density SNP genotyping chips are considered. GWAS analysis requires a large sample size to achieve sufficient statistical power [91,92]. Attaining a large population size for tick resistance studies is a challenge in developing countries, because of lack of funds, resources, well trained data collectors and infrastructure. To tackle this problem, the establishment of collaborations could be beneficial, since it will promote the sharing of information, insights and funds. This will improve the development of GWAS and its quality. 

### 7.4. Data Quality Control for GWAS

Prior to actual genome-wide association analysis, the genotyped data is subjected to quality control (QC) to decrease the chances of discovering false positive and false negative associations [93]. False positive association is defined as the occurrence of identifying a SNP association that is not truly influencing the trait of interest in the study. On the other hand, false negative association is defined as an incidence where a SNP that is influencing the trait of interest is not associated with the trait in the study. The genotyped data is subjected to stringent filters that are performed on a sample and SNP level. The quality control filters include missing call rate, minor allele frequency (MAF) and Hardy Weinberg equilibrium (HWE). The samples with a missing call rate higher than 1–5% could be a result of poor DNA quality potentially influencing genotypic errors [56]. The removal of SNPs with high missing genotypes may increase SNPs with accurate genotype calls for downstream analysis. However, in a study with a small population size, it is not good practice to lose samples because this negatively affects the power of the study. This can be overcome through genotype imputation to replace the missing SNP markers, explained later. 

The other aspect of QC is to assess the format of genotyped data, where sometimes there is a mixing of the AB and ACTG formats, which needs to be corrected to form one uniform format. Additionally, data is assessed for MAF, which removes SNPs not complying with a given threshold for a particular study. Very low allele frequencies are less informative [94] and can result in the discovery of fake associations [95]. GWAS capitalizes on the LD that exists between the markers; thus, it is very important to assess the deviation of SNPs from the HWE. The deviation of SNPs from HWE is set using Chi square test [96], where the significance level for rejecting is based on P values ranging from 10^−5^–10^−7^ [50,56]. The SNPs that are not in compliance with a stipulated criterion are removed from the dataset that is used in the downstream analysis. Overall, statistical software such as PLINK, and R environment are used for quality control. However, PLINK is the most preferred because it is free and flexible to accommodate large scale data management [56]. It is noted there is no universal criterion to perform QC and there is no perfect QC pipeline that is able to capture all the problematic SNPs in a population being studied. Therefore, it is important to view the clustering intensity plots for SNPs, to ensure that there are no obvious clustering problems.

### 7.5. The Extent of LD Measures r^2^ in GWAS 

GWAS relies on the extent of LD between markers across the genome, where its measure (r^2^) is bounded between 0 and 1, with 1 considered as the perfect association [48]. Linkage disequilibrium in a population can be affected by population structure, genetic drift, selection, recombination rate, migration and mutations [97]. The development of LD maps and haplotype block structures at the population level are useful parameters for guiding GWAS. In association studies, the presence of LD creates two possible outcomes, namely, direct and indirect association [98]. Direct association occurs when the SNP influencing a biological system is directly genotyped in the study and found to be statistically associated with the trait. The indirect association outcome occurs when the SNP is indirectly linked with the trait. The phenomenon is termed taq SNP and is graphically explained well in a review by [98]. The feasibility of GWAS strongly depends on the extent of LD, as the latter determines the required SNP markers and mapping resolution [99]. Therefore, it is important to study the extent of LD in a population of interest before the association analysis is performed. 

### 7.6. The Effect of Genotype−Environment Interaction

According to [100], genotype by environment (GxE) interaction is defined as the different responses of genotypes under different environmental conditions. GxE affects the ranking of animal performance under different environmental conditions. Therefore, it is important to closely monitor economical traits that are largely influenced by environmental factors [101]. In cattle production, tick resistance is among the traits that are highly influenced by environmental factors. Climate change influences the distribution and density of tick populations and, because it has adverse influences on the life cycle of ticks, it increases the chances of tick−host interactions.

Currently, genetic evaluations for tick tolerance in countries such as Australia and Brazil are performed routinely. Despite the success of these evaluations, ref. [102] noted that GxE is not taken into consideration in these evaluations. They also pointed out that failure to consider GxE interaction in genetic evaluations can potentially affect genetic gain, as the selection of candidate comparisons is environmentally dependent. Thus, the cost implications of not accounting for GxE can be high [103]. This is because animals observed as top performers in one environment will not necessarily perform the same in a different environment, a phenomenon that is associated with the loss of genetic progress. Few studies have provided evidence that resistance to ticks in cattle can be influenced by various environmental effects [104]. A recent study investigated the existence of GxE using different models in Hereford and Braford cattle [102]. Their findings showed that the estimates of repeatability varied along the environmental gradient (range 0.18–0.45), indicating that resistance to ticks is environmentally influenced. Additionally, the posterior means of the genetic correlations across the environmental tick infestation surface plot demonstrated a large plateau above 0.80. This indicates that there will be re-ranking of performance for a trait of interest between environments, which necessitates the separation of breeding programs for each environment [105,106]. GxE interaction contributes to the genetic architecture of complex traits and it affects the chances of discovering a true association between phenotype and genotype in GWASs. According to [107], failure to adjust for environmental effects results in the reduced chances of predicting an association. This has been proven through assimilation studies, intensively in human epidemiological studies [108,109,110] and plant studies [111,112]. 

### 7.7. Batch Effect

For most study designs, samples are not genotyped at once, instead, they are handled in batches. One of the reasons behind this can be the use of large number of samples, which makes it impossible to genotype the samples at the same time. In addition, some studies collect samples at different time intervals, prompting the genotyping to be also conducted at different time intervals. For cattle tick studies, sometimes data is collected from different environments that are geographically spaced, leading to data being treated in batches. Additionally, for cattle ticks studies conducted on hybrids, the data is partitioned according to the development of the hybrids. It is known that the development of hybrid populations can never be carried out at one go. Instead it is conducted in batches, for example, the development of an F2 population. The partitioning of samples gives rise to the batch effect, which results in apparent associations confounded by the batches. It is therefore necessary to assess the dataset for a potential batch effect, since it has potential to yield spurious association if this is not accounted for. Before the actual GWAS association test, the batches’ genotyped data should be handled independently to assess the presence of confounding effects [113].

### 7.8. Genotype Imputation as a Cost Effective Approach to Improve the Power of GWASs

The main purpose of imputation is to infer missing genotypes of the SNPs that are not directly genotyped in the study, using *in silico* haplotype information from reference samples with genotypes from denser genotyping arrays [114,115]. Genotype imputation holds the potential to improve the statistical power to detect association by reducing the number of missing genotypes, thereby increasing the overall number of genotypes available for association analysis [114]. Genotype imputation has the potential to boost GWAS statistical power by up to 10% over testing only genotyped SNPs [116]. The performance of association tests on typed SNPs may not lead to a significant association, especially when the sample size is small [114]. Their findings were different when the association test was conducted on imputed genotyped data, where there was also a more detailed view of the association region. 

Various tools are available for imputation and some are presented in Table 4. For example, BEAGLE has been built to handle genotype intensity data so that genotypes can be called using LD information between the SNPs, offering an improvement in genotyping error rates. Imputation is a cost effective tool to generate genomic variants at denser platforms [46,117]. Imputation allows high density genotypes to be imputed reliably from low density SNP arrays [117]. This potentially solves the affordability issue in developing countries, as more animals can be genotyped at low cost. However, the accuracy of imputation and the factors that affect it should be taken into consideration because they determine the reliability of the tool. 

In humans, whole genome sequencing and imputation based GWAS strategies were used to refine the association signals and recover novel association signals for complex traits [118,119]. Sequencing and imputation GWAS is powerful and cost effective, and can also be applied on non-European populations [120]. In cattle, the whole genome sequencing and imputation GWAS strategies have been applied to study the genetic architecture of quantitative traits in beef cattle [50]. In relation to tick resistance, most GWAS were carried out using 50K genotyping platforms (Table 5). Thus, some studies used imputation to ensure better statistical analysis effectiveness [13,14,121]. Imputation boosted the number of SNPs that were tested for association and subsequently improved the power. Imputation makes it applicable for cattle tick resistance-focused studies to be conducted using samples that were initially genotyped at low density platforms, then imputed to high density. This has been proposed as a cost-effective approach than can solve the current problems associated with generating genomic data in cattle production, especially in developing countries. Currently, in different regions, there are ongoing studies that are investigating the feasibility of the tool for cattle research. The outcomes of these studies will give insights that could be used to properly apply this tool in GWAS aimed to study biological mechanisms underlying tick resistance.

## 8. Progress on Tick Resistance GWAS in Cattle

Quantitative trait loci studies using microsatellites and SNPs have been inconsistent, with a very low percentage relating phenotypic variation to tick infestation [122]. Most studies were conducted in subtropical regions such as Brazil, Australia and Mexico (Table 5). Brazilian studies have successfully mapped genomic regions associated with resistance to ticks on F_2_ Gyr × Holstein and on Hereford and Braford [13]. Similar study were conducted in Australia by [123]. In some instances, different studies identified QTLs on similar chromosomes, regardless of the differences in the breeds and tick species used. This underlines the need for the validation of the role of these chromosomes in cattle tick resistance. Validation could be pursued through GWAS meta-analysis, which can be achieved through collaborations. 

To date, only one study in Africa used SNP genotyping and GWAS as an approach to investigate genetic variants associated with tick resistance in cattle [8]. This study was conducted on South African Nguni cattle and identified several genomic regions harboring QTLs associated with tick count traits. Despite the studies conducted previously, information on genetic determinants associated with cattle resistance to ticks is still limited. Further investigations focusing on unravelling genomic determinants associated with tick resistance will identify and provide understanding on biological mechanisms associated with TTBDs in cattle production. The information from the investigations will present a great opportunity to improve breeding programs to produce animals that are more resistant to tick infestation, while enhancing productivity [7,12].

## 9. Breeding Cattle for Tick Resistance

Breeding for genetic resistance is a potentially promising strategy to control ticks [124]. Sufficient genetic variation is one of the key factors determining the success of breeding schemes in livestock production. Bovine quantitative genetics studies have demonstrated low to high heritability for resistance to ticks depending on the breed [125,126]. Such findings hold the potential for tick resistance to be included as a goal in breeding schemes. *Bos taurus* breeds are known to be highly productive; however, they are also known to be highly susceptible to ticks and this makes their use in tropical production systems unsustainable, especially by resource-poor farmers. On the other hand, *Bos indicus* breeds are known for being resilient toward ticks as compared to *B. taurus*, but they yield lower production. 

These differences motivate the development of crossbreeding programs between *Bos indicus* and *Bos taurus* cattle. These programs aim to produce crossbred animals, to facilitate the improvement of production whilst controlling TTBDs through the use of genomic selection. The success of genomic selection for tick resistance depends on the availability of proper genetic evaluation programs for the trait. The existence of genetic evaluation programs for tick resistance has the potential to generate information and facilitate the improvement of tick resistance. In countries such as Australia and Brazil, there are ongoing genetic evaluation programs for tick resistance. These develop crossbred animals that are productive under environmental conditions that have a high prevalence of ticks [127]. In Brazil, there is a genetic evaluation program known as Delta G Connection, which involves the Hereford and Braford cattle [102]. The Australians have developed the Australian Friesian Sahiwal, which produces acceptable levels of milk in an environment with ticks. The lack of information of such programs from other regions may suggest less enthusiasm from other regions, especially Africa and Asia [127]. There are concerns about the selection potential for resistance to ticks and the tradeoffs with other traits of economic significance. In this regard, studies have demonstrated that there is very low genetic correlation between tick count and various productive, adaptive and pubertal traits [105]. 

The existence of genetic components of variation in host resistance to ticks in cattle is currently being studied to discover tick resistance molecular markers that can be used in marker assisted selection (MAS). This information from locally adopted breeds can be used through crossbreeding to upgrade local breeds using highly productive exotic breeds. The challenges hindering the application of MAS for tick resistance are costs, resource populations, requirements of technical skills and the validation of discovered molecular markers for each population. All these challenges translate to the lack of molecular markers associated with tick resistance in cattle. However, despite the limitations, the development of studies that will investigate and generate genomic information on tick resistance and production traits holds the potential to increase the accuracy of selection. Therefore, the use of molecular genetics techniques, together with conventional breeding tools, is important in balancing the process of selection for tick resistance. 

Host resistance to ticks is potentially an alternative tick control strategy that could solve the current TTBDs problems affecting the beef and dairy industry. The development of this alternative strategy requires the generation of knowledge that will broaden the understanding of biological mechanisms underlying tick resistance in cattle. Generally, the first step to control for a certain trait of interest requires the studying of genetic determinants influencing the expression of the trait. The advancement of technology has made it possible to use a genome-wide association approach to gain understanding on mechanisms underlying tick resistance in cattle and to generate knowledge. However, information is still limited, and this is one of the hurdles preventing the facilitation of breeding for tick resistance through genomic selection. The information gap warrants the need for more GWASs to be conducted, to provide an understanding on biological mechanisms underlying tick resistance.

The costs associated with TTBDs are a major constrain for beef and dairy production. Therefore, the use of genomic selection as a tool to breed for tick resistant cattle will reduce the costs associated with the intensive use of vaccines and acaricides. This will further ensure animal welfare, facilitate increased production and subsequently increase profit margins. The implementation of genomic selection for tick resistance in developing countries is currently hindered by the high costs associated with the generation of a large training population, phenotyping and genotyping. The success of genomic selection for tick resistance is possible through the use of cost-effective genotype imputation methods to increase the power of GWASs and the accuracy of GEBVs estimates. There are countries that have included tick resistance trait in their breeding goals. However, from those countries, there are no studies that have been put in place to investigate the economic aspects of including the tick resistance trait in a genomic selection breeding goal. Therefore, there is a need for studies that will investigate the prospect of cost-effective genomic selection for tick resistance.

## 10. Limitations for GWAS to Uncover Tick Resistance Causal Variants in Cattle

The discovery of common causal variants associated with tick resistance is limited by various attributes, which include the nature of the trait, methodological challenges and the lack of financial resources. Tick resistance is a polygenic trait, influenced by more than one gene. This means the discovery of major genes through the use of GWAS is impossible. Factors such as epistasis, epigenetics, microbiome, and environment attribute to missing heritability [128,129]. This results in GWAS not being able to capture all the genetic determinates underlying the expression of tick resistance trait [14]. The lack of biological understanding on how these factors influence missing heritability in cattle is part of the obstacles hindering the discovery of true loci associated with tick resistance. The failure of GWAS to capture common variants is not a limitation to GWAS only. To date, there is no perfect genomic technology available to capture all the genetic information underlying the expression of complex traits [130]. Despite this drawback, there is a pressing need to use the available technologies to discover information that could be used to improve the trait through selection. Methodological challenges such as the GWAS study design of choice, poor structure of the data, phenotyping uncertainties, genotyping errors, improper data analysis, and the poor interpretation of results plays a huge role in limiting the success of GWAS for the trait of interest. The use of a poor data structure and failure to ensure proper data analysis increase the discovery of type I and type II errors. Therefore, ensuring proper data analysis and a clear interpretation of results is crucial for the generation of information that will inform alternative controlling strategies for ticks. The fluctuating exchange rate makes it difficult for most researchers to conduct GWAS studies focused on studying the genetic architecture of tick resistance, since such studies require a large sample size that is expensive to develop, sustain and genotype.

## 11. Conclusions

When conducting GWAS, it is necessary to account for factors that affect the rate of discovering an association, and control the rate of discovering spurious associations. GWASs have been very successful in the discovery of SNPs and candidate genes associated with tick resistance in various cattle breeds of different origins. Despite the success, more information is needed given that most GWASs on tick resistance in cattle are underpowered. This underlines the need for continuous data collection to enable larger and more powerful studies. For the studies that have been conducted using low density markers, genotype imputation is the most appropriate cost-effective approach for GWAS for tick resistance in cattle. The availability of modest research populations, tools and funds are the current limitations of GWASs in developing countries. GWASs in African countries are performed using SNP genotyping arrays developed using exotic breeds. Thus, ascertainment bias effect leads to a low discovery rate of variants that influence the expression of the phenotype. This underscores the need for the inclusion of information from local breeds of different regions in the development of SNP genotyping arrays for cattle. This will facilitate a better understanding of variation in the breeds that are naturally adapted to the African production environments.

## Figures and Tables

**Table 1:** Common publicly available computer programs for GWAS.

Software	Focus	Website	Reference
PLINK	Stratification, LD and structured association mapping	http://pngu.mgh.harvard.edu/purcell/plink, accessed on 20 May 2019	[34]
R (GenABEL)	Stratification, LD and structured association mapping	https://cran.r-project.org/src/contrib/Archive/GenABEL, accessed on 20 May 2019	[35]
SVS	Stratification, LD, haplotype blocs and structured association mapping	http://www.goldenhelix.com, accessed on 1 June 2019	[36]
GenAMap	Stratification, LD and structured association mapping	http://cogito-b.ml.cmu.edu/genamap, accessed on 1 June 2019	[37]
GEMMA	Stratification, Fits LMM and BSLM models, IBD analysis, estimation of chip heritability, and association mapping.	http://www.xzlab.org/software.html, accessed on 1 June 2019	[38]
Blupf90	Data conditioning, estimate variances using several methods, and use SNP information for improved accuracy of breeding values + for genome-wide association studies (GWAS)	http://nce.ads.uga.edu/wiki/doku.php?id=documentation, accessed on 17 November 2021	[39]

GEMMA—genome-wide efficient mixed model association; LLM—linear mixed model; BSLM- Bayesian sparse linear mixed model; SNPs—single nucleotide polymorphisms; LD—linkage disequilibrium; IBD—identical by descent; SVS—SNP and variation suite.

**Table 2:** Some web databases that house genomic information associated with economic traits.

Genomic Database	Description	URL
NCBI (Genbank)	Repository for biomedical and genomic information	https://www.ncbi.nlm.nih.gov/ accessed on 6 December 2019
Ensembel	Genome browser	https://www.ensembl.org/index.html accessed on 6 December 2019
Animal QTLdb	Animal QTL database	https://www.animalgenome.org/cgi-bin/QTLdb/index accessed on 4 September 2020
NAGRP	Genomic information browser	https://www.animalgenome.org/ accessed on 4 June 2020
EMBL-EBI	Genomic information database	https://www.ebi.ac.uk/ accessed on 15 June 2020
DDBJ	Genomic information browser	https://www.ddbj.nig.ac.jp/index-e.html accessed on 15 June 2020
UCSC	Genome browser	https://genome.ucsc.edu/ accessed on 15 June 2020
Refseq	Reference sequence database	https://www.ncbi.nlm.nih.gov/refseq/ accessed on 15 June 2020
VEGA	Genome browser	http://vega.archive.ensembl.org/index.html accessed on 15 June 2020

Animal QTLdb—Animal quantitative trait loci database; NAGRP—national animal genome research program; EMBI-EBI—European molecular biology laboratory-European bioinformatics institute; DDBJ—DNA data bank of Japan; UCSC—University of California Santa Cruz; Refseq—Reference sequence; VEGA—vertebra genome annotation.

**Table 3:** Models that can be used for GWAS analysis.

Model Type	Model	Reference
Single locus	General linear model(GLM)	[62]
	Mixed lieanr model (MLM)	[63]
	Logistic mixed model(LMM)	[64]
	Compressed mixed linear model (CMLM)	[63]
Multi-locus	Multilocus random SNP effect mixed linear models(mrMLM)	[65,66]
	Fast multilocus random SNP effect effiecient mixed model association (FASTmrEMMA)	[67]

**Table 4:** Some available software packages for genotype imputation.

Software	Usage	Website
BEAGLE	Prephases haplotypes infers missing genotypes, and identifies IBD in related samples	https://Faculty.washington.edu/browning/beagle/old.beagle.html accessed on 9 July 2020
GIGI	Imputes missing genotypes on a pedigree	https://faculty.washington.edu/wijsman/progdists/gigi/software/GIGI/GIGI.html accessed on 9 July 2020
IMPUTE2	Prephases haplotypes, infers missing genotypes	https://mathgen.stats.ox.ac.uk/impute/impute_v2.html accessed on 9 July 2020
MaCH/minimac3	Prephases haplotypes, infers missing genotypes	https://github.com/statgen/Minimac4 accessed on 9 July 2020

IBD—identical by descent; GIGI—Genotype imputation given inheritance.

**Table 5:** Previous GWAS studies on genomic regions associated with tick resistance in different regions of the world.

Region	Breed	Sample Size	Mode of Infestation	Genotyping Platform	Findings	Reference
Brazil	F_2_ *B. taurus* × *B. indicus*	382	Artificial	Microsatellite	Identified significant genomic regions on chromosomes 5, 7 and 14	[11]
Brazil	F_2_ Gyr × Holstein	376	Artificial	Microsatellite markers	Identified dry season specific QTL on BTA 2 and 10, rainy season specific QTL on BTA 5, 11 and 27 and BTA 23 for both seasons	[12]
Australia	Brown-Swiss, Holstein-Friesian, mixed taurine	189	Natural	MegAllele genotyping bovine10K SNP	Identified genes associated with tick burden, namely *TNFSF8 [CD30]*, and *SIRPA*	[123]
South Africa	Nguni	586	Natural	Illumina BovineSNP50 BeadChip	Identified significant genomic regions on chromosomes 1, 3, 6, 7, 8, 10, 11, 12, 14, 15, 17, 19 and 26	[8]
Brazil	Braford and Hereford	3455	Natural	Illumina BovineSNP50 BeadChip	Identified 48 tag SNPs associated with tick resistance	[13]
Brazil	F_2_ Gir × Holstein	46	Artificial	Illumina BovineSNP50 BeadChip	Identified genes associated with immune system function, namely, *TREM1*, *TREM2*, *CD83*, *MYO5A*, *TREML1*, and *PRSS16*	[14]

F_2_—Second filial generation; QTL—quantitative trait loci; BTA—Bos taurus; SNPs—single nucleotide polyimorphisms.
